# Preventing mother to child transmission of HIV: lessons learned from China

**DOI:** 10.1186/s12879-020-05516-3

**Published:** 2020-10-26

**Authors:** Yu Dong, Wei Guo, Xien Gui, Yanbin Liu, Yajun Yan, Ling Feng, Ke Liang

**Affiliations:** 1 Department of Geriatrics, Zhongnan Hospital of Wuhan University, Wuhan, China; 2 Department of Pathology, Wuhan University School of Basic Medical Sciences, Wuhan, China; 3 Department of Pathology, Zhongnan Hospital of Wuhan University, Wuhan, China; 4 Department of Infectious Diseases, Zhongnan Hospital of Wuhan University, 169 Donghu Road, Wuhan, 430071 China; 5 Center of Preventing Mother-to-child transmission for Infectious Diseases, Zhongnan Hospital of Wuhan University, 169 Donghu Road, Wuhan, 430071 China; 6 Department of Nosocomial Infection Management, Zhongnan Hospital of Wuhan University, Wuhan University, Wuhan, Hubei China

**Keywords:** Antiretrovirals, HIV, Mother-to-child transmission, Prevention, Trend

## Abstract

**Background:**

The program for the prevention of mother-to-child transmission (PMTCT) of human immunodeficiency virus (HIV) was launched in 2003 in China, but few studies have been conducted to describe the panorama of PMTCT. We investigated the rate and associated factors of mother-to-child transmission (MTCT) in China from 2004 to 2018.

**Methods:**

HIV-infected pregnant women from two areas in China between 2004 and 2018 were enrolled. Antiretrovirals (ARVs) were provided to the mothers and their babies, and the children were followed and tested for HIV.

**Results:**

In total, 857 mothers and their 899 children were enrolled, and the overall MTCT rate was 6.6% (95% CI 5.0–8.2). The MTCT rates of nonintervention, only formula feeding (FF), infant prophylaxis (IP) + FF, single dosage antiretrovirals (sdARVs) + IP + FF, zidovudine (AZT) alone+IP + FF and prenatal combination antiretroviral therapy (cART) + IP + FF were 36.4, 9.4, 10.0, 5.7, 3.8 and 0.3%, respectively. The MTCT rate declined over time. No ARVs, CD4 count < 200/μL, low birth weight, and breastfeeding were associated with MTCT of HIV. For different ARVs, a higher MTCT rate was observed for AZT alone, sdARVs, and no ARVs compared to cART for pregnant women.

**Conclusions:**

Although the overall MTCT rate remains relatively high, the real-world effect of prenatal cART+IP + FF in China has exerted the same protective effects in high-income countries. With the extension of prenatal cART for pregnant women with HIV, the MTCT rate of HIV has gradually declined in China. However, the coverage of prenatal cART for pregnant women should be further improved. The effect of only post-exposure prophylaxis for infants was limited.

## Background

Over the past twenty years, the prevention of mother-to-child transmission (PMTCT) for human immunodeficiency virus (HIV) has achieved significant successes worldwide. The recommendations of the World Health Organization (WHO) on the regimens of antiretrovirals (ARVs) for pregnant women infected with HIV have evolved significantly over time, from single dosage antiretrovirals (sdARVs) and zidovudine (AZT) alone (2004) to lifelong combination antiretroviral therapy (cART), regardless of the patient’s immune status (2013) [1–3]. The strategy of cART for pregnant women evolved from option A to option B to option B+ [4]. However, PMTCT displays a disparate effect globally due to imbalanced health resource distribution. In high-income countries, mother-to-child transmission (MTCT) rates have decreased to less than 1% [5, 6]. For example, only 44 HIV-positive infants were born in the United States in 2016, with an estimated incidence of 1.1/100,000 live births [7]. In eastern and southern Africa, the MTCT rate was still as high as 9% in 2018 [8]. Therefore, measures should be pursued to improve PMTCT in settings with limited resources, especially in low- and middle-income countries.

In China, although PMTCT for HIV program was launched in 2003, the MTCT rate was still as high as 5.7% in 2016 [9]. In addition, most published studies about PMTCT in China have focused on the effect of PMTCT over short periods or one of antiretroviral regimen for pregnant women [10, 11], and little attention has been paid to describing the panorama of PMTCT in China and exploring existing questions. In addition, since China has adopted PMTCT strategies for a long time [12–14], knowing the effects of these strategies over different time periods is essential.

This study aimed to investigate the real-world effects of PMTCT on HIV over time (2004–2018) in two areas of China and to evaluate the risk factors for PMTCT. The reasons for the relatively high MTCT rate were preliminarily investigated, which could benefit relevant policies.

## Methods

### Participant recruitment

As an extension of earlier work [15, 16], HIV-positive pregnant women who were confirmed HIV positive during antenatal care from two areas (Hubei Province, recruited during January 2004 and December 2018; Yining in Xinjiang Uygur Autonomous Region, recruited during January 2004 and December 2012) of China were enrolled. HIV-positive pregnant women with terminated pregnancies, pathological pregnancies, loss to follow-up, and children whose follow-up was not completed at the endpoint were excluded.

Furthermore, if a woman had more than one pregnancy during the study, each pregnancy was treated as a separate event. Multiple births were also treated as separate events.

### Data collection

Related information, including demographics, intrapartum CD4+ T lymphocyte count (CD4 count), and data about HIV-positive mothers and their children were collected via medical charts from hospitals and the AIDS Comprehensive Prevention and Control Data Information Management System of the Chinese Center for Disease Control and Prevention (CDC). The reasons why the pregnant women missed cART were investigated in interviews and by checking associated information. Children’s follow-ups were conducted in cooperation with local CDC and Maternal and Child Health Hospitals.

### Regimens of ARVs for PMTCT

The regimens of ARVs for pregnant women and their babies were in accordance with the constantly updated recommendations from both the WHO [1–3] and China’s [12–14] guidelines and the timing of being diagnosed with HIV infection. Thus, the regimens of ARVs for pregnant women were categorized as 1) prenatal cART: cART before delivery; 2) AZT alone: only AZT before delivery; 3) single sdARVs: sdARVs at delivery; and 4) no ARVs: no ARV agent was used before or at delivery.

Furthermore, infant prophylaxis (IP) included single dosage nevirapine (sdNVP), sdNVP and AZT for 1 week, and NVP or AZT for 4–12 weeks.

Thus, the measures were classified as 1) prenatal cART+IP + formula feeding (FF); 2) AZT alone+ IP + FF; 3) sdARVs+IP + FF; 4) IP + FF; 5) only FF; 6) nonintervention; and 7) other measures: ARVs+FF without IP, ARVs+IP without FF, and only IP.

### Follow-up and confirmation of MTCT

The babies were followed up at the 1st, 3rd, 6th, 9th, 12th, and 18th months after birth. Proviral DNA of HIV was tested at the 6th week and the 3rd month, and enzyme-linked immunosorbent assay (ELISA) of HIV antibodies were examined determined at the 12th and 18th months. Children’s HIV status was defined according to the national HIV diagnostic criteria [17]. In general, for children older than 18 months old, positive ELISA and western blot were adopted to diagnose HIV infection. For children younger than 18 months old, two positive proviral DNA tests were adopted as the criterion to diagnose HIV infection, and negative ELISA or two negative proviral DNA tests defined HIV-negative status. The date for the endpoint of the follow-up was December 2018.

### Statistical analyses

The results are described as percentages and 95% confidence intervals (CIs). The median maternal age was used to define the cutoff point for converting numerical variables into dichotomous variables. Either Pearson’s chi-square test or Fisher’s exact test was used to compare categorical variables between the HIV-infected and uninfected children groups. Furthermore, multilevel logistic regression was performed to assess the factors associated with PMTCT. The use of a multilevel model was justified by the children being grouped by area and birth year. We modeled the probability [adjusted odds ratio (AOR)] of MTCT as a function of ARVs for pregnant women, ARV regimens for pregnant women, intrapartum CD4 count, mode of delivery, birth weight, IP, breastfeeding, and complex interventions, adjusting for the area, year of birth, maternal age, ethnicity, route of infection, HIV status of sexual partner, multipara or not, and sex of the infant. Moreover, the trend was analyzed by the Cochran Armitage test. All of the analyses were conducted with SAS software, version 9.4.

## Results

### The mothers and children enrolled in the study

**Fig. 1:**
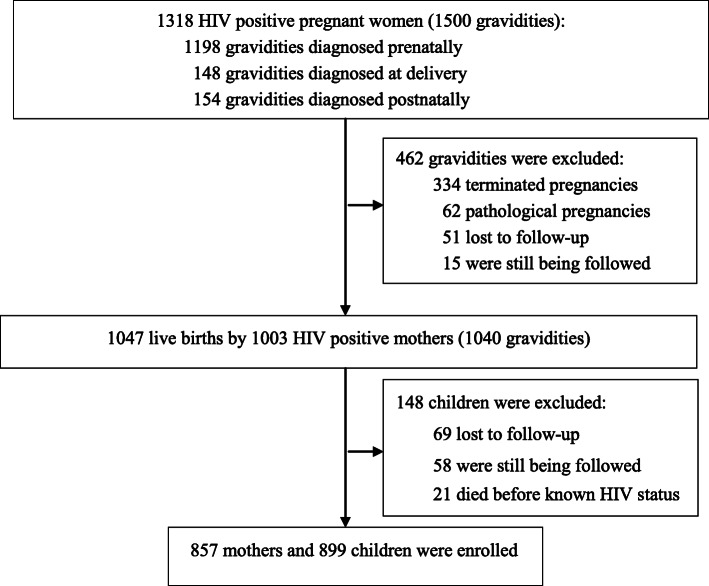
Derivation of the study population

In total, 1318 HIV-infected pregnant women (1500 gravidities) were identified, and 462 gravidities were excluded (334 terminated pregnancies, 62 pathological pregnancies, 51 losses to follow-up, 15 still being followed at the endpoint of the study). Overall, 1047 children were liveborn, and 148 children were excluded (69 were lost to follow-up, 58 were being followed at the endpoint of the study, and 21 died before diagnosis). A total of 857 mothers and 899 children with known HIV status were enrolled in this study (Fig. 1). The median age of the pregnant women was 27 years old (range 17–48 years old).

### Effects of PMTCT on HIV

**Fig. 2:**
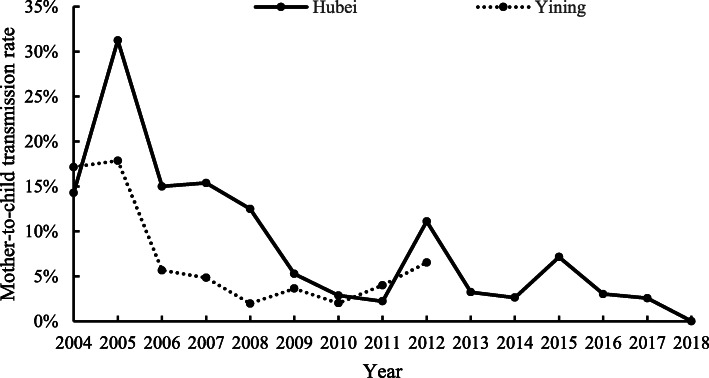
Trends of MTCT rate in two areas of China, 2004–2018

The overall MTCT rate was 6.6% (95% CI 5.0–8.2) among the 899 children. From 2004 to 2018, the MTCT rate declined significantly year by year in both areas. For Hubei Province, the MTCT rate decreased from 26.1% in 2004–2005 to 1.5% in 2017–2018 (Z = -4.00, P for trend < 0.001). For Yining, the MTCT rate decreased from 17.5% in 2004–2005 to 5.2% in 2011–2012 (Z = -2.67, P for trend = 0.008) (Fig. 2).

The complex measures of prenatal cART+IP + FF were extended, and both AZT alone +IP + FF and sdARVs+IP + FF were replaced gradually, especially in Hubei. However, the MTCT rate was still as high as 3.4% in 2014–2018 (cART was recommended by the WHO from 2013 for all pregnant women who were prenatally diagnosed with HIV infection [3]), and only 78.7% of mother-child pairs received prenatal cART+IP + FF, while 9.6% received IP + FF, 8.4% received only FF, and 3.4% were on nonintervention during 2014–2018.

During 2014–2018, 38 pregnant women did not receive prenatal cART in Hubei Province, while 24 (63.2%) of them were postnatally diagnosed with HIV infection, and 14 (36.8%) of them refused to take ARVs.

The MTCT rate in pregnant women with ARVs (prenatal cART, AZT alone or sdARVs) + IP + FF was 2.7% (18/658), which was significantly lower than that in the IP + FF group (10.0%) (Fisher’s exact test, *P* = 0.011). The MTCT rates for nonintervention, only FF, IP + FF, sdARVs+IP + FF, AZT alone+IP + FF, and prenatal cART+IP + FF were 36.4, 9.4, and 10.0% and 5.7, 3.8 and 0.3%, respectively. According to the results, the more comprehensive that the measures taken were, the lower that the MTCT rate achieved was (Z = -9.16, P for trend < 0.001). The MTCT rate did not differ significantly between the only FF group and the IP + FF group (Fisher’s exact test, *P* = 1.000). (Table 1).

**Table 1:** Social-demographic and status of mothers and children in two areas of China, 2004–2018 (*N* = 899)

	Total number	HIV-infected	MTCT rate	χ^**2**^	***P***
	(n = 899)	(***n*** = 59)	(%)(95%CI)		
**Maternal age (years)**				0.164	0.686
≤ 27	480	33	6.9 (4.7–9.2)		
> 27	419	26	6.3 (4.0–8.6)		
**Areas**				0.358	0.549
Hubei	469	33	7.1 (4.8–9.4)		
Yining	430	26	6.1 (3.8–8.3)		
**Ethnicity**				..	0.483
Han	457	32	7.0 (4.7–9.3)		
Uygur	412	24	5.8 (3.6–8.1)		
Others	30	3	10.0 (2.1–26.4)		
**Route of infection**				..	0.188
Sex	857	54	6.3 (4.7–7.9)		
Others^a^	42	5	11.9 (4.0–25.6)		
**HIV status of sexual partner**				..	0.836
Negative	466	29	6.2 (4.0–8.4)		
Positive	362	26	7.2 (4.5–9.8)		
Unknown	71	4	5.6 (0.3–11.0)		
**Multipara or not**				0.068	0.793
Yes	365	23	6.3 (3.8,8.8)		
No	534	36	6.7 (4.6,8.9)		
**ARVs for pregnant women**				62.442	< 0.001
Yes	666	18	2.7 (1.5–3.9)		
No	233	41	17.6 (12.7–22.5)		
**Regimens of ARVs for mothers**				69.053	< 0.001
Prenatal cART	333	1	0.3 (0.0–1.7)		
AZT alone	81	3	3.7 (0.8–10.4)		
sdARVs	252	14	5.6 (2.7–8.4)		
No ARVs	233	41	17.6 (12.7–22.5)		
**Intrapartum CD4 count(/μL)**				29.782	< 0.001
≥ 200	491	15	3.1 (1.5–4.6)		
< 200	78	14	17.9 (9.4–26.5)		
Unknown	330	30	9.1 (6.0–12.2)		
**Mode of delivery**				2.468	0.116
Cesarean section	544	30	5.5 (3.6–7.4)		
Vaginal delivery	355	29	8.2 (5.3–11.0)		
**Sex of infant**				..	0.795
Female	407	25	6.1 (3.8–8.5)		
Male	452	31	6.9 (4.5–9.2)		
Unknown	40	3	7.5 (1.6–20.3)		
**Birth weight**				..	< 0.001
≥ 2500 g	772	41	5.3 (3.7–6.9)		
< 2500 g	55	11	20.0 (9.4–30.6)		
Unknown	72	7	9.7 (2.9–16.6)		
**IP**				56.327	< 0.001
Yes	720	25	3.5 (2.1–4.8)		
No	179	34	19.0 (13.3–24.7)		
**Breastfeeding**				..	< 0.001
Never	831	34	4.1 (2.7–5.0)		
Ever	68	25	36.8 (25.3–48.2)		
**Complex interventions**				..	< 0.001
Prenatal cART+IP + FF	331	1	0.3 (0.0–1.7)		
AZT alone+IP + FF	80	3	3.8 (0.8–10.5)		
sdARVs+IP + FF	247	14	5.7 (2.8–8.6)		
IP + FF	60	6	10.0 (2.4–17.6)		
Only FF	106	10	9.4 (3.9–15.0)		
Non-intervention	66	24	36.4 (24.8–48.0)		
Others	9	1	11.1 (0.3–48.0)		

^a^others include 32 cases of blood route (paid plasma donation and blood transfusion), 9 cases of intravenous drug, and 1 case of MTCT

.. no statistic value obtained by Fisher’s exact test

The MTCT rates were 0% (0/125), 0% (0/122), 1.2% (1/86), and 5.0% (3/60) when cART was adopted before conception or during the 1st trimester, during the 2nd trimester, during the 3rd trimester, and intrapartum, respectively. The MTCT rate decreased significantly as pregnant women took cART earlier (Z = -2.95, P for trend =0.003).

There was no significant difference in the MTCT rate between cesarean sections and vaginal deliveries among the 331 children with prenatal cART+IP + FF (0.38%, 1/265 vs. 0, 0/66, Fisher’s exact test, *P* = 1.000).

### Risk factors for MTCT

The results of multilevel logistic regressions showed that, compared to no ARVs, the MTCT rate for pregnant women with ARVs (prenatal cART, AZT alone or sdARVs) was significantly lower (AOR = 0.11, 95% CI: 0.06, 0.23). For different complex interventions, the multivariate results indicated that the MTCT rate for AZT alone+IP + FF, sdARVs+IP + FF, IP + FF, only FF, and nonintervention was higher than that for prenatal cART+IP + FF. CD4 count ≥200/μL was less likely to cause MTCT compared with CD4 count < 200/μL (AOR = 5.41, 95% CI: 2.32, 12.61).

The MTCT rate did not differ significantly between the only FF (9.4%) and IP + FF (10.0%) groups (AOR = 0.99, 95% CI: 0.32, 3.06). Cesarean section did not reduce the risk of MTCT compared with vaginal delivery (*P* = 0.227), but breastfeeding presented a significantly higher MTCT rate than formula feeding (AOR = 12.31, 95% CI: 5.78, 26.20). Babies with lower birth weight (< 2500 g) showed a higher risk (AOR = 5.18, 95% CI: 2.27, 11.81) of HIV infection. (Table 2).

**Table 2:** Factors associated with MTCT of HIV in two areas of China, 2004–2018 (N = 899)

	AOR^a^ (95% CI)	*P*	AOR^b^ (95% CI)	*P*
**Maternal age (years)**				
≤ 27	1		1	
> 27	0.96 (0.54, 1.71)	0.894	0.89 (0.47, 1.66)	0.696
**Ethnicity**				
Han	1		1	
Uygur	0.38 (0.03, 4.36)	0.413	0.38 (0.03, 4.58)	0.423
Others	0.69 (0.09, 5.09)	0.702	0.69 (0.09, 5.27)	0.706
**Route of infection**				
Sex	1		1	
Others	1.13 (0.36, 3.58)	0.818	1.14 (0.34, 3.82)	0.825
**HIV status of sexual partner**				
Negative	1		1	
Positive	1.15 (0.60, 2.21)	0.671	1.16 (0.60, 2.24)	0.649
Unknown	0.77 (0.22, 2.63)	0.664	0.83 (0.23, 3.01)	0.765
**Multipara or not**				
Yes	1.17 (0.64, 2.16)	0.594	1.19 (0.61, 2.30)	0.598
No	1		1	
**Sex of infant**				
Female	1		1	
Male	1.10 (0.62, 1.97)	0.736	1.12 (0.62, 2.00)	0.703
Unknown	0.74 (0.19, 2.94)	0.661	0.88 (0.20, 3.79)	0.858
**ARVs for pregnant wome**n				
Yes	0.14 (0.07, 0.27)	< 0.001	0.11 (0.06, 0.23)	< 0.001
No	1		1	
**Regimens of ARVs for mothers**				
Prenatal cART	1		1	
AZT alone	12.87 (1.18, 140.65)	0.037	11.35 (1.03, 124.80)	0.047
sdARVs	14.23 (1.61, 126.12)	0.018	13.27 (1.48, 118.83)	0.022
No ARVs	56.02 (7.01, 447.60)	< 0.001	62.33 (7.77, 500.15)	< 0.001
**Intrapartum CD4 count(/μL)**				
≥ 200	1		1	
< 200	5.21 (2.28, 11.87)	< 0.001	5.41 (2.32, 12.61)	< 0.001
Unknown	3.13 (1.42, 6.92)	0.006	3.41 (1.53, 7.59)	0.004
**Mode of delivery**				
Cesarean section	1		1	
Vaginal delivery	1.41 (0.76, 2.62)	0.263	1.46 (0.78, 2.75)	0.227
**Birth weight**				
≥ 2500 g	1		1	
< 2500 g	4.93 (2.20, 11.02)	< 0.001	5.18 (2.27, 11.81)	< 0.001
Unknown	1.11 (0.44, 2.78)	0.818	1.33 (0.42, 4.15)	0.619
**IP**				
Yes	1		1	
No	5.76 (3.04, 10.93)	< 0.001	6.74 (3.43, 13.23)	< 0.001
**Breastfeeding**				
Never	1		1	
Ever	11.14 (5.38, 23.03)	< 0.001	12.31 (5.78, 26.20)	< 0.001
**Complex interventions**				
Prenatal cART+IP + FF	1		1	
AZT alone+IP + FF	13.86 (1.29, 149.25)	0.031	12.21 (1.13, 131.71)	0.040
sdARVs+IP + FF	16.17 (1.84, 142.00)	0.013	15.05 (1.71, 132.86)	0.015
IP + FF	34.53 (3.88, 307.51)	0.002	35.81 (4.00, 320.54)	0.002
Only FF	31.77 (3.74, 270.02)	0.002	35.99 (4.22, 306.99)	0.001
Non-intervention	162.32 (19.56, 1347.36)	< 0.001	179.81 (21.59,1497.57)	< 0.001
Others	39.02 (1.94, 783.13)	0.017	40.21 (1.98,817.95)	0.017

*AOR* Adjusted odds ratio

AOR^a^ were adjusted for area and year of birth

AOR^b^ were adjusted for area, year of birth, maternal age (years), ethnicity, route of infection, HIV status of sexual partner, multipara or not, and sex of infant

## Discussion

Knowing the risk factors and temporal trends of MTCT was essential for further improving the effect of PMTCT. This study demonstrated the real-world effects and associated factors of PMTCT for HIV in two areas of China and extended the existing literature [10, 11]. The panorama of PMTCT and existing questions in enrolled areas were exhibited, providing more evidence for formulating relevant policies in China.

In this study, the MTCT rate in the nonintervention group was 36.4% (95% CI 24.8–48.0), which was in concordance with the result of our previous retrospective study (34.8%) [18]. This finding is also comparable with the results of studies conducted before the adoption of interventions to reduce vertical transmission, which ranged between 13 and 48% in South and Southeast Asia [19]. With the progress of technology in China, the MTCT rate declined year by year. These results demonstrate a remarkable reduction in HIV vertical transmission through the extended application of PMTCT in China. However, the total MTCT rate (3.4%) during 2014–2018 was still relatively high compared with the target of zero new infections among infants [20]. There is still much to do to further reduce MTCT in China since the MTCT rate in the UK has already declined to less than 1% [6], and MTCT of HIV has been eliminated in Cuba, Thailand, and Belarus [21]. We also analyzed the reasons for pregnant women not receiving prenatal cART in 2014–2018. The study showed that 63.2% of them missed prenatal HIV testing although the government provided free prenatal HIV screening. They received their first HIV test on admission to the hospital for delivery, and the results came later than the baby was born. In contrast, 36.8% of them refused cART. Based on our experience, the reasons included the fear of revealing HIV infection or the stereotype that taking pregnancy drugs was harmful to the fetus. Thus, the importance of prenatal HIV screening and cART should be widely emphasized. Moreover, more health education about the effects of PMTCT and how to manage HIV infection correctly should be provided for HIV-infected pregnant women.

Our study demonstrated that ARVs (prenatal cART, AZT alone, or sdARVs) for pregnant women with IP + FF could reduce the MTCT rate to 2.7% -- significantly lower than only IP + FF (*P* = 0.011). Although AZT alone and sdARVs for pregnant women can significantly reduce the MTCT rate compared with only IP + FF (3.8 and 5.7% vs. 10.0%), the MTCT rate of prenatal cART+IP + FF was less than 1/10 that of AZT alone+IP + FF (0.3% vs. 3.8%) in our study, in accordance with findings from another study (1.2% vs. 10.4%) [22]. Our results indicated that the real-world effect of prenatal cART+IP + FF in China had the same effects as PMTCT in high-income countries (< 1%) [5, 6]. The results of multilevel logistic regression in this study also indicated that prenatal cART was associated with the lowest risk of MTCT, and the risk of MTCT in pregnant women with CD4 < 200/μL was 5.4 times that in those with CD4 ≥ 200/μL. Furthermore, the study reported that MTCT rates decreased rapidly with each additional week of cART for pregnant women, especially within 15 gestational weeks [23]. Similarly, there was a downward trend for the MTCT rate regarding longer duration of cART by the time of delivery in our study. Additionally, the study proposed that maternal cART might improve pregnant women’s health, in turn promoting child survival by improving the mother’s ability to care for the child [24]. Thus, HIV-infected pregnant women should receive cART as early as possible due to its apparent effect on PMTCT and its benefits for maternal and child health.

Our study also indicated that breastfeeding is an independent risk factor for MTCT. This finding is consistent with previous studies. For example, a clinical trial conducted in Kenya suggested that avoiding breastfeeding could reduce the MTCT rate by 44% (from 36.7 to 20.5%) [25]. Our study also reported that formula feeding decreased the MTCT rate by 74% (from 36.4 to 9.4%). Although some data have indicated that there was no MTCT of HIV by breastfeeding for mothers with virologic suppression [26], in settings in which safe and affordable feeding alternatives exist, breastfeeding is not typically recommended for mothers with HIV. Due to the significant effect of formula feeding on reducing the MTCT rate, up to 92.4% (831/899) of children received formula feeding, and none of the children who received formula feeding died during our follow-up, suggesting that formula feeding is a feasible and essential intervention for PMTCT of HIV in China.

The WHO recommends that HIV-exposed infants receive prophylaxis, whether they are breastfed or formula fed [3]. A clinical trial indicated that IP extended until the end of breastfeeding led to a very low postnatal MTCT rate [27], suggesting the importance of IP. However, the effect of only post-exposure prophylaxis (PEP) on the prevention of intrapartum HIV transmission has rarely been studied due to ethics concerns. Our study can answer this question in the condition of conforming ethics. We found that there was no significant difference in the MTCT rate between the IP + FF and only FF groups, and by multilevel logistic regression analysis, IP + FF was not a protective factor against PMTCT compared with FF alone. The latest study in Switzerland also suggested that PEP can be avoided for newborns if the mother is virologically suppressed, sparing infants the toxicity associated with ARVs [28]. These results suggested that the effect of only PEP for infants was limited if the pregnant women did not receive ARVs. Therefore, cART for pregnant women and formula feeding are more critical for reducing MTCT than IP.

In our study, the MTCT rate did not vary between cesarean section and vaginal delivery in children with prenatal cART+IP + FF. Poor evidence has suggested that elective cesarean section appears to reduce infant HIV acquisition but increase maternal and infant morbidity [29]. Furthermore, the WHO does not recommend elective cesarean section in resource-limited settings, specifically for HIV infection [30, 31]. Cesarean section brings a substantial economic burden for pregnant women. However, a large proportion of HIV-infected pregnant women with prenatal cART+IP + FF chose cesarean delivery in our study (80.1%, 265/331), so knowledge about cesarean section in the context of maternal cART should be imparted to health workers and pregnant women, and unnecessary cesarean section should be avoided.

An investigation reported that low birth weight was an independent risk factor for MTCT [32], and our study revealed similar findings, indicating that HIV-exposed infected infants were more likely to have low birth weight than HIV-exposed uninfected infants. Moreover, infants with low birth weight had higher morbidity of HIV in the neonatal period than other infants [33]. Notably, lower birth weight by uterine cART exposure in uninfected infants was rapidly corrected during early infancy [34]. Therefore, prenatal cART should be administered to reduce not only vertical HIV transmission but also low birth weight.

Several limitations of our study must be acknowledged. First, 21 babies who died before receiving HIV testing were excluded, which might have induced selection bias and underestimated the MTCT rate in our study. Among the babies who died, ten babies were in sdARVs+IP + FF group, four babies were in the IP + FF group, six babies were in the only FF group, and one baby was in the nonintervention group. Moreover, a few other HIV-related parameters that could play a role in predicting MTCT were not measured (such as viral loads) or were incomplete (such as CD4 count) in this cohort, which could have biased our results.

## Conclusions

Even with these limitations, we still concluded that the MTCT rate gradually declined over time in the two study areas, while cART was considered to be an important strategy for preventing MTCT, and the coverage of prenatal cART should be further improved. While the overall MTCT rate was still relatively high in the two enrolled areas, strategies that aim to consider the findings from this study and further improve the effects of PMTCT are required.

## Data Availability

The datasets used and/or analysed during the current study are available from the corresponding author on reasonable request.

## Abbreviations

HIV: Human immunodeficiency virus
MTCT: Mother-to-child transmission
PMTCT: Prevention of mother-to-child transmission
ELISA: Enzyme-linked immunosorbent assay
PCR: Polymerase chain reaction
ARVs: Antiretrovirals
AZT: Zidovudine
NVP: Nevirapine
sdARVs: Single dosage antiretrovirals
sdNVP: Single dosage Nevirapine
IP: Infant prophylaxis
FF: Formula feeding
cART: Combination antiretroviral therapy
CDC: Centers for Disease Control and Prevention
PEP: Post-exposure prophylaxis
